# Alterations of HPV-Related Biomarkers after Prophylactic HPV Vaccination. A Prospective Pilot Observational Study in Greek Women

**DOI:** 10.3390/cancers12051164

**Published:** 2020-05-05

**Authors:** George Valasoulis, Abraham Pouliakis, George Michail, Christine Kottaridi, Aris Spathis, Maria Kyrgiou, Evangelos Paraskevaidis, Alexandros Daponte

**Affiliations:** 1 Department of Obstetrics & Gynaecology, University Hospital of Larisa, Mezourlo, 41334 Larisa, Greece; dapontea@otenet.gr; 2 Hellenic National Public Health Organization-ECDC, Marousi, 15123 Athens, Greece; 3 Second Department of Pathology, Attikon University Hospital, National and Kapodistrian University of Athens, Chaidari, 12462 Athens, Greece; apou1967@gmail.com (A.P.); ckottaridi@gmail.com (C.K.); arisspa@gmail.com (A.S.); 4 Department of Obstetrics & Gynaecology, University Hospital of Patras, Rio, 26504 Patras, Greece; gr.michail@yahoo.com; 5 West London Gynaecological Cancer Center, Department of Obstetrics & Gynaecology, Queen Charlotte’s and Chelsea Hospital, Hammersmith Hospital, London W120HS, UK; mkyrgiou@yahoo.com; 6 Department of Obstetrics & Gynaecology, University Hospital of Ioannina, Neochoropoulo, 45500 Ioannina, Greece; vangelispar@hotmail.com

**Keywords:** HPV vaccine, colposcopy clinic, HPV DNA genotyping, HPV mRNA E6 and E7, pilot study, persistent HPV infection

## Abstract

The objective of this study was to investigate the hypothesis that HPV vaccination administered in patients with low-grade (LG) cytology shortly after an initial colposcopic assessment could prospectively alter HPV-related biomarkers. This was a prospective pilot observational study involving women attending a colposcopy clinic for evaluation of abnormal LG cytology that were advised to undergo HPV vaccination and proceeded accordingly. These women were compared with a matched unvaccinated group. Women requiring cervical biopsies or CIN treatment were excluded. Intervention: A full three-dose HPV vaccination was undertaken with either the 2-valent or the 4-valent anti-HPV VLP vaccine. LBC samples were obtained prior and after the completion of the vaccination regimen and tested for HPV DNA genotyping (CLART-2 HPV test) and E6 and E7 mRNA (NASBA technique). Results: Alterations of HPV-related biomarkers at a colposcopy reassessment appointment 12 months later. Analysis: The *p*-values, relative risk (RR), absolute relative risk (ARR), number needed to treat (NNT) and 95% confidence intervals for each biomarker in each group were assessed. Results: A total of 309 women were included in the analysis. One hundred fifty-two women received the vaccine. HPV vaccination reduced in a statistically significant manner (*p* < 0.05) HPV DNA positivity rates for genotypes 16, 18, and 31, RR = 1.6 (95% CI: 1.1 to 2.3), RR = 1.7 (95% CI: 1.1 to 2.8), and RR = 1.8 (95% CI: 1.0 to 2.9), in women who only tested DNA-positive for HPV16, 18, and 31 genotypes, respectively, prior to vaccination. A less pronounced, statistically insignificant reduction was shown for women who tested positive for both HPV DNA and mRNA E6 and E7 expression for HPV16, 18, and 33 subtypes. Statistically significant reduction in HPV mRNA positivity was solely documented for genotype 31 (*p* = 0.0411). Conclusions: HPV vaccination appears to significantly affect the rates of HPV16, 18, and 31 DNA-positive infections in the population testing HPV DNA-positive for the aforementioned genotypes. The above findings deserve verification in larger cohorts.

## 1. Introduction

The consistent implementation of cervical cancer screening programs in the mid-1980s led to significant reductions in this neoplasm’s morbidity and mortality rates in several developed countries. More recently, the global launch of prophylactic HPV vaccination programs materialized primary cervical cancer prevention and empowered the aspiration of a possible disappearance of cervical cancer in the near future [1,2]. However, cervical cancer screening and HPV vaccination programs are not being applied worldwide in a universal manner. Across Europe, large disparities are still observed between affluent and poorer countries in terms of primary and secondary cervical cancer prevention strategies and infrastructure.

In Greece, despite several initiatives, no national cervical cancer screening program is currently in place, and most women are being opportunistically screened, predominantly by cytology and lately by HPV DNA and mRNA co-testing. Individuals with cytological abnormalities are referred for further assessment mainly to specialized colposcopy centers. Commencing in 2007, Greece was among the first countries to incorporate the anti-HPV vaccine (2-valent and 4-valent virus-like particle (VLP) vaccines) free of charge in the national immunization program. Throughout the study period, a vaccination strategy was ongoing with a primary cohort being adolescent girls from 12 to 15 years of age, while a catch-up cohort of older females up to the age of 26 was discontinued in December 2016 with the introduction of the 9-valent HPV vaccine. However, of note is that no vaccination registry or any other similar system is in place in Greece to monitor vaccination uptake; all relevant information is based on unpublished data from the Ministry of Health. Therefore, it seems that only approximately 30–35% of the eligible target group individuals have received the full regimen until today. There are several reasons for this disappointing coverage; predominantly, an active anti-vaccine lobby and ineffective counseling by physicians [3].

The aim of this study was to investigate possible alterations of HPV DNA and mRNA E6 and E7 biomarkers’ expression of oncogenic HPV genotypes HPV16 and 18, as well as of the oncogenic HPV genotypes genetically related to HPV16/18, i.e., HPV31, HPV33, and HPV45, after prophylactic HPV vaccination with 2-valent and 4-valent VLP vaccines in individuals who adhered to the advice provided at the participating colposcopy clinics and received the vaccine (vaccinated population (group V)) compared to those who did not (matched controls (non-vaccinated Group– [Group N-V])). The preliminary results of a study of population subgroups with different inclusion criteria were presented at the annual meeting of the British Society for Colposcopy and Cervical Pathology, BSCCP, in 2013 [4].

## 2. Results

As stated, the main purpose of the study was to examine the evolution of documented single HR HPV infections in individuals who subsequently received the HPV vaccine in comparison to the controls who did not. A total of 309 women were included to the final analysis of the study, subdivided in 20 separate subgroups in total based on the HPV subtype at the initial assessment (HPV DNA genotyping).

The first ten subgroups represented the study population for the first analysis of HPV DNA-positive infections, in which mRNA was tested negative. The remaining 10 subgroups represented the population subgroups for the second analysis, where both HPV DNA- and mRNA-positive infections were present (positive) at the initial assessment. We analyzed the data for HPV genotypes 16, 18, 31, 33, and 45. The distribution of women in the abovementioned subgroups is presented in Table 1.

### 2.1. Demographic Data

One hundred ninety-two (192) women with HPV DNA-positive and mRNA-negative infections and 117 women with both HPV DNA- and HPV mRNA-positive infections were enrolled. One hundred fifty-two women received the vaccine, and all of them completed the three-dose scheme within 7 months from the enrolment. The administration ratio of the two regimens was 64% for the 2-valent to 36% for the 4-valent anti-HPV vaccine, respectively. This phenomenal disproportion was mainly attributed to the fact that the 2-valent vaccine’s (Cervarix™) price was almost half of the corresponding 4-valent’s (Gardasil4™) price at the Greek market throughout the study period. The mean age of the studied population was 26.5 years (range: 18–35 years). Twenty-five percent of those women had given birth, and 52% of them were current smokers. The mean age of onset of sexual intercourse was 17 years and 45% of the women had had more than 3 sexual partners.

Respectively, the 157 women of the non-vaccinated control group had a mean age of 26.6 years, 26.1% of them had given birth, and 47.1% of them were current smokers. The mean age of onset of sexual intercourse was 17 years and 38.5% of the women had had more than 3 sexual partners (see Table 2). Neither demographic population characteristic was statistically significant neither between the vaccinated and non-vaccinated populations nor between the mRNA-positive and mRNA-negative groups (see *p*-values, Table 2).

### 2.2. Outcomes

#### 2.2.1. Observed Alterations of HPV16 DNA Expression

Among the 24 women in the V1 group (for the definition of study groups and subgroups please see Section 4.1 and Section 4.4) who had solitary HPV16 DNA infections at the initial assessment, following administration of the vaccine, only four still tested positive for HPV16 DNA at the 12-month molecular evaluation, whereas in the N-V1 group (N = 28), 13 individuals were found to express HPV16 DNA at that time (see Table 3). Therefore, the women who received the regimen appeared to have statistically significant superior clearance rates (83% vs. 54%) in comparison to the women who did not (*p* = 0.043).

Regarding the HPV DNA- and mRNA-positive infections in the V2 and N-V2 groups (see Table 3), the administration of the vaccine appeared to reduce HPV DNA positivity rates. Specifically, the data analysis showed that among the 12 women who had been vaccinated, 50% (6/12) had cleared the HPV infection compared to 31% (4/13) in the N-V2 group approximately 5–6 months after the 3^rd^ dose of the vaccine (at the 12-month evaluation) (*p* = 0.567).

#### 2.2.2. Observed Alterations of HPV16 mRNA

None of the women in the V1 group appeared to have progression of the disease in terms of the first testing positive for mRNA E6 and E7 after vaccination, while among the women who did not receive the vaccine, 3 out of the 13 who remained HPV16 DNA-positive (11% of the N-V1 population) also tested positive for HPV16 mRNA E6 and E7, suggesting persistent infection (*p* = 0.0985).

A remarkable finding was the possible effect of the vaccine on elimination of initially mRNA-positive infections, where 67% of the cases in the V2 group regressed in terms of mRNA E6 and E7 expression. Out of the 12 recruited women, 6 retested HPV DNA-positive and 4 tested mRNA E6- and E7-positive at the 12-month evaluation (*p* = 0.3019).

#### 2.2.3. Observed Alterations of HPV18 DNA Expression

Among the 25 women who fulfilled the criteria for recruitment tested positive for HPV18 and were included in the V1 group, 6 (24%) retested positive for HPV18 DNA at the 12-month re-evaluation. At that time, 56% (14/25) of women in the N-V1 group were found harboring persistent HPV18 infection (see Table 3). Therefore, women who received the regimen demonstrated a 76% clearance rate in comparison with 44% (11/25) of the women who did not (N-V1 group) (*p* = 0.043).

Regarding the populations of the V2 and N-V2 groups, among the 13 women with HR HPV18 DNA- and mRNA-positive infections at recruitment, just 6 (46%) tested positive after the anti-HPV vaccine administration versus 60% (12/20) in the N-V2 group. Therefore, the regimen appeared to have a positive effect on HPV18 DNA and mRNA-positive infections in terms of turning 54% of them HPV DNA-negative, although in a non-significant manner (*p* = 0.672) (Table 3).

#### 2.2.4. Observed Alterations of HPV18 mRNA Expression

At the 12-month reassessment, out of the 6/25 HPV DNA-positive individuals in the V1 group, one also tested positive for mRNA E6 and E7. Thus, in spite of vaccination, one individual (corresponding to 4% of the group population) had evidence of molecular progression of the HPV18 disease (positive DNA- and mRNA E6 and E7 tests). Regarding women that had not been vaccinated (N-V1 group), out of the 14 women who were found with persistent infection (in terms of the positive HPV DNA test at the 12-month evaluation), 3 (12% of the N-V1 population) progressed and also tested positive for HPV18 mRNA E6 and E7 (*p* = 0.2971).

Similarly, notable was the effect of the vaccine on elimination of mRNA E6 and E7 expression in the V2 and N-V2 study groups. Ten out of the 13 individuals (77%) in the V2 group tested negative for HPV18 mRNA E6 and E7 at the 12-month visit, while 6 of them remained HPV DNA-positive at that time. Respectively, only 35% of the individuals who did not receive the regimen tested positive for HPV18 mRNA E6 and E7 at the 12-month visit.

#### 2.2.5. Observed Alterations of HPV31 DNA Expression

Among the 28 women in the V1 group who were tested HPV31-positive at the initial assessment, only 6 (21%) appeared to have a persistent HPV infection and tested positive for HPV31 DNA at the 12-month evaluation. On the contrary, in the N-V1 group (N = 20), 11 individuals (55%) were found to be still HPV31 DNA-positive (see Table 3). Therefore, the women who received the regimen appeared to have a 79% clearance of the infection in comparison to 45% of the women who did not (*p* = 0.036).

Regarding the HR HPV31 DNA- and mRNA-positive infections, in the V2 group (see Table 3), the administration of the vaccine appeared to reduce HPV DNA positivity to 53% at the 12-month visit in comparison to the non-vaccinated population (N-V2 group), where HR HPV31 DNA regression was found in 38% of the cases. Detailed data analysis showed no evidence of statistically significant differences (*p* = 0.879).

#### 2.2.6. Observed Alterations of HPV31 mRNA Expression

Three out of the 28 initially mRNA E6- and E7-negative women (corresponding to 11%) in the V1 group appeared to be mRNA E6- and E7-positive at the 12-month evaluation despite vaccination, while in the unvaccinated population (N-V1 group), 7 out of the 11 women who remained HPV DNA-positive (12 months after recruitment) also tested positive for HPV31 mRNA E6 and E7, expressing a molecular progression of the disease. Detailed data analysis of that subgroup revealed that HPV vaccination led to a statistically significant reduction of mRNA E6 and E7 expression (*p* = 0.0411).

Regarding the V2 group, out of the 8 HPV DNA-positive individuals at the 12-month evaluation, 4 women (27%) remained positive for mRNA E6 and E7. Out of the non-vaccinated women (N-V2 group), eight (50%) remained HPV mRNA E6- and E7-positive, while, as mentioned earlier, 10 women from this subgroup remained HPV DNA-positive (*p* = 0.1826) (Table 3).

#### 2.2.7. Observed Alterations of HPV33 and HPV45 Expression

Out of the 18 women in the V1 group who initially tested HPV33 DNA-positive, only 28% had persistent HPV33 DNA infection upon reassessment. Out of the 17 women positive for HPV33 DNA that did not receive the regimen (N-V1), 47% had persistent infection. The comparison of the HPV DNA regression rates between these two groups did not reach statistical significance (*p* = 0.407) (Table 3). Similarly, statistically insignificant differences were observed between the two groups of women expressing both HR HPV33 DNA and mRNA E6 and E7 (V2 and N-V2) (*p* = 0.859) (see Table 3).

Additionally, the alterations of the HPV33 mRNA expression between the two paired subgroups (V1 and N-V1 as well as V2 and N-V2) did not reach statistical significance.

Regarding the HPV45 expression, the small number of women (10 individuals) harboring this genotype precluded definitive conclusions.

#### 2.2.8. Overall Results for Vaccinated vs. Non-Vaccinated Women

When the studied population was assessed as two groups, vaccinated vs. non-vaccinated women, irrelevant of the HPV mRNA status and the HPV subtype at the end of the study period, 85 individuals (54.1%) from the group of non-vaccinated women (N = 157) remained HPV DNA-positive. At the same time (end of the study period), out of the corresponding 152 women of the vaccinated population, 47 (30.9%) re-tested HPV DNA-positive. Thus, the non-vaccinated women exhibited inferior HPV DNA clearance rates (*p* < 0.0001, OR: 0.38, 95% CI: 0.24–0.60). The power analysis that was conducted to evaluate statistical significance of these results resulted in 98.4%, indicative of a low error probability (Table 4).

In the mRNA-positive cohort (N = 117) at enrolment, 42.2% (N = 27 out of 64) of the non-vaccinated women re-tested positive, while only 32.1% of the vaccinated women (N = 17 out of 53) remained mRNA-positive at the end of the study (*p* = 0.2610) (Table 5). In contrast to the results of the HPV DNA clearance, in which the calculated power exceeded 95%, the power of the results regarding HPV mRNA clearance was only 16.6%.

In conclusion, for all HPV genotypes, the percentage of HPV DNA clearance was higher for the women that were vaccinated, irrespective of whether this was reflected in positivity solely for HPV DNA or for both HPV DNA and HPV mRNA. HPV45 represented an exception because of the limited number of cases (see Table 3). Statistically significant differences were observed for HPV16, HPV18, and HPV31 genotypes and only for the groups of mRNA E6 and E7-negative viral infections.

## 3. Discussion

Today, almost fifteen years after the launch of the first-generation prophylactic anti-HPV VLP vaccination, the existing literature remains controversial over the potentially augmented HPV clearance in prevalent HPV infections and pre-existing dysplasias precipitated by these vaccines. Studies have illustrated that the endogenous response towards HPV infection is less robust in magnitude and/or duration compared to the immune response elicited by the first-generation VLP vaccines [5] with these being able to stimulate 10- to 100-fold higher levels of the L1-specific serum neutralizing antibodies compared to those of the natural infection [6]. Our prospective study is among the first to document improved outcomes in terms of HPV16 and 18 clearance rates over time among women with established minor cervical dysplasias who have received the anti-HPV vaccine post-exposure.

Close to the launch of the first-generation VLP vaccines, Hildesheim et al. published their results of a randomized trial which they considered evidence of lack of a therapeutic effect of 4-valent–based vaccination on pre-existing HPV infections [7]. The two study’s arms (VLP vaccine and placebo) examined more than 800 individuals each (participating in the Costa Rica Vaccine trial) approximately 11 months after enrolment and receiving the first 2-valent dose. No major differences in viral clearance and vaccine efficacy for viral clearance for HPV16 and HPV18 in the study group at 6 and 12 months of follow-up were observed among the two groups. The authors did acknowledge that they could not rule out the possibility that vaccination prevents progression to cyto- or histological outcomes, as well as that women with established infections might benefit from vaccination in other ways at the individual level. However, they concluded that even if vaccination provides some benefit, it is likely small and therefore clinicians should discourage use of L1 VLP-based vaccines in the context of prevalent pre-existing CIN lesions. Finally, they commented that it is unclear whether vaccination would benefit women with prevalent infection by increasing their immunologic resistance against reappearance of the same viral type in the future, as the case would be if T cell responses developed following an initial HPV infection that successfully clears failed to eradicate subsequent viral infections.

In a relatively similar Dutch population setting, Mollers et al. prospectively followed a large cohort of 14–16 y.o. girls, half of whom had been vaccinated, for three years [8]. The authors concluded that vaccination is effective against incident and persistent infections with HPV16/18 and HPV16/18/31/45. The importance of vaccination before sexual debut was highlighted by the lower vaccine efficacy against persistent HPV16/18 infection in the girls positive at the baseline.

Studies of mid-adult women have illustrated that incident high-risk HPV detection is most likely attributed to either probable new acquisition or redetection of a prior infection [9]. In this light, based on the VLP vaccine’s properties, active immunization of colposcopy patients might not only permit a swift discharge from the colposcopy clinic, but could further prevent them from returning to colposcopy in the course of reactivation of a latent infection or a new re-infection by a different HPV genotype. The impact of the above highlights is even more substantial given that in the fall of 2018, the FDA finally granted approval for the 9-valent HPV vaccine in women of the expanded 26–45 y.o. group [10]. The broadening of the age range for HPV vaccination candidacy has for years underpinned the X. Bosch’s HPV-FASTER strategy [11], a project with robust rationale that, however, necessitated vast financial resources and infrastructures besides political will.

The importance of HPV vaccination of previously HPV-infected women is also substantiated in a recent publication by Vorsters et al., who consider this practice safe and generating a high-level immune response [12]. The authors contend that even in women with a productive infection, vaccination will lead to a potentially neutralizing amount of transudated anti-HPV antibodies in their cervicovaginal secretions with several benefits: first, vaccination could prevent infectious virions from a productive infection spreading from sites with low potential for malignant progression to the cervical transformation zone with a higher potential for progression. Additionally, the vaccine might decrease the likelihood that women with a productive infection transmit the infection to their sexual partner and therefore will no longer (or to a lesser extent) be able to re-infect themselves or transmit their infection(s).

Several studies have so far assessed the effects of anti-HPV vaccine administration in patients following cervical conization [13,14,15]. All these studies have documented favorable outcomes for individuals who received the anti-HPV vaccine following definitive cervical pre-cancer treatment, with fewer CIN relapses for the vaccinated arms. More recently, the Speranza study [16] has been one of the largest published prospective case–control studies of HPV vaccination following local CIN excisional treatment. Patients were followed up for a prolonged 4-year period, and disease relapse was established by histology. The study illustrated that the full three-dose vaccination course with the 4-valent vaccine shortly after loop electrosurgical excision procedure (LEEP) conization showed an impressive 80% clinical effectiveness in disease relapse prevention. Interpreting the results, the authors concluded that despite the fact that HPV vaccines have no therapeutic effect on the prevalent HPV infection or disease, it might be beneficial as an adjuvant additional to surgical treatment. A larger RCT studying woman aged 18–55 with biopsy-confirmed high-grade CIN is currently being conducted in the UK (the NOVEL trial, NCT03979014).

Our study aimed to examine the impact of VLP vaccination on cervical infections caused specifically by HPV genotypes 16, 18, 45, 31, and 33. In part, this was dictated by the implementation of the NASBA methodology which detects transcripts of those five high-risk HPV genotypes. Furthermore, as robust data illustrate, these particular genotypes are responsible for the vast majority of high-grade cervical pre-cancer and cervical cancer cases in Greece [17,18]. Based on the design of our study, we did not include individuals who underwent conization or cervical biopsies, since HPV-related biomarker expression can be affected by cervical interventions, both therapeutic [19] and diagnostic [20]. However, from a practical standpoint, vaccinating individuals who are undergoing cervical biopsies might indeed prove beneficial, possibly to a lesser extent than that observed in patients undergoing conization, in which an increased viral load is removed with the cone; this notion should obviously be corroborated with prospective randomized studies.

The correlation of HPV mRNA positivity with persistence of cervical HPV infection is another finding that emerged from our study, as statistically significantly fewer CIN-affected patients with positive mRNA assays managed to clear positivity for 16/18 following vaccination; this valuable information gives insight on which patients might benefit more from receiving this vaccine. HPV mRNA assays represent useful triage tools which are able to differentiate cervical lesions with a true premalignant potential [21,22]. With HPV integration and overexpression of E6/E7 heralding genomic instability, HPV infection is less likely to regress and high-grade lesions are likely to develop over time.

The high percentage of women who received the 2-valent vaccine in our study (64% for the 2-valent vaccine vs. 36% for the 4-valent one) might provide a plausible explanation regarding the high rates of effectiveness by cross-protection mechanisms observed against infections caused by HPV31 and HPV33. Data from the end-of-study analysis of PATRICIA illustrated consistent vaccine efficacy against persistent infection and CIN2+ (with or without HPV16/18 co-infection) across cohorts for HPV33, HPV31, HPV45, and HPV51 [23]. Furthermore, a recent publication [24] suggested that cross-protection is higher in 2-valent vaccine recipients compared to 4-valent vaccine recipients. These findings are in line with the recent Cochrane review [25] which also showed similar protection of 2-valent and 4-valent vaccines against cervical pre-cancers related to types 16/18, but concluded that the 2-valent vaccine offered better protection against cervical pre-cancers overall. From an immunogenicity perspective, several studies have so far reported significantly higher antibody levels against vaccine and non-vaccine types elicited by the 2-valent vaccine compared to 4-valent one [26,27,28].

Despite the observed statistical power of DNA clearance rates for all the studied HPV subtypes was high (98.4%), a major limitation of this study was the low relevant power for mRNA clearance, reaching only 16.6%. This is attributed to the limited number of cases in the cohort of mRNA-positive women; out of the 309 HPV DNA-positive women, only 117 women simultaneously expressed HPV mRNA. As the study focuses on smaller subgroups, statistical power drops are inevitable, especially for patients harboring less prevalent genotypes (i.e., HPV33 and 45). Based on our study population, we calculated that 680 women would be required to achieve statistical significance for HPV31 DNA clearance and more than 1200 women for the corresponding clearance of HPV31 mRNA; even higher numbers would be required for genotypes 33 and 45. The studies focusing on these specific genotypes’ HPV DNA or mRNA persistent infection detection should be designed with a high number of participants ad hoc.

Awaiting results of larger studies, it could be suggested that all the women harboring cervical dysplasias can be advised to receive the full course of the anti-HPV vaccine irrespective of age and whether they are being managed under surveillance or have undergone definitive conservative surgery. Additionally, the combined analysis of readily obtainable HPV-related biomarkers (either with cytological or self-sampling samples) [29,30,31,32,33,34] could promote their incorporation either in screening and/or decision-making prior to colposcopy, as well as in the follow-up period in patients with minor (low-grade) or severe (high-grade) cytological abnormalities, enabling the risk-based individualized surveillance/treatment plan [21,35,36]. This consultation must be strong and straightforward, given that in several settings, vaccine administration will be individually-funded (at a considerable cost in countries with average GDP or high vaccine price, as was the case in our study). Conclusively, our study suggests that despite the fact that the current prophylactic anti-HPV vaccines do not possess therapeutic properties, it is plausible that under certain circumstances they might be helpful in enhancing natural HPV infection clearance rates.

## 4. Materials and Methods

### 4.1. Study Population

A prospective pragmatic observational study was designed to include women who were referred to and attended the colposcopy clinics of the University Hospitals of Patras, Ioannina, Attikon, Athens, and colposcopy clinic of the Larisa Primary Health Center, Greece, for further assessment and evaluation of abnormal cytology. The colposcopy clinic of the Larisa Health Center refers to the University Hospital of Larisa. Similarly, the colposcopy clinic of the Ioannina University Hospital is the referral center of an extensive territory covering north-west Greece and several Ionian Islands, while the corresponding clinic of the University Hospital of Patras covers the remainder of north-west Greece, the Peloponnese, and Ionian islands. Finally, the Athens University’s 3^rd^ Ob/Gyn Department (Attikon Hospital) serves extensive less affluent areas of the Greek capital. Experienced gynecologists and board-accredited colposcopists staff the aforementioned departments.

The strong recommendation for HPV vaccination represents universal standard clinical policy for non-vaccinated individuals in all the aforementioned participating clinics. Non-vaccinated women who got vaccinated after consultation during a colposcopic evaluation comprised vaccination group V; these were compared with a similarly referred group who did not proceed with vaccination for some reason, mainly financial constraints (group N-V), despite an identically worded consultation. All women were informed about the scope of the study and were asked to sign a consent form before entering the study. The study’s protocol was approved by the Greek Central Government (Ministry of Education and Religious Affairs) under the frame of the HPVGuard research project [37] (http://HPVGuard.org, project number: 11ΣΥΝ_10_250, cooperation framework, protocol number: ΕΥΔΕ – ΕΤAΚ 1788/1-10-2012) and subsequently received additional approval from the coordinating authority—the Attikon University Hospital Ethics Committee (code: ΕΒΔ 623/14-5-13).

### 4.2. Inclusion and Exclusion Criteria

Unvaccinated young women up to the age of 35 with cytological and/or colposcopic evidence of abnormal cytology (ASCUS+ to low-grade disease (low-grade squamous intraepithelial lesions [LGSIL])) who tested positive for HPV DNA genotyping (measured by the CLART-2 HPV test, Genomica, Spain) for any one of the HPV16, 18, 31, 33, and 45 genotypes (as solitary infections) and either positive or negative for mRNA E6 and E7 as tested by the NASBA technique (measured by the NucliSENS EasyQ test, bioMerieux) at the initial visit were included. All the specimens were centrally analyzed at the Attikon General University Hospital of Athens.

We excluded individuals with cytological and/or colposcopic evidence of high-grade disease, women who had been scheduled for cervical biopsies or cervical intraepithelial neoplasia (CIN) treatment, women who tested positive for the HPV genotypes different from the above mentioned ones, and women who at the time of recruitment and initial evaluation were found to have incident HPV16, 18, 31, 33, and/or 45 co-infection. Women who did not complete vaccination on time (within 7 months from the recruitment) were excluded.

For the purposes of this particular analysis, we included individuals who were recruited in a 4.5-year span, from November 2012 until April 2017, shortly after the launch of the 9-valent vaccine.

### 4.3. Study Protocol

Based on the study protocol, in all the women, extensive gynecological history was obtained at the first visit. Detailed specific history regarding data on HPV immunization, age at coitarche, number of sexual partners since coitarche, and the use of condoms; in addition to the epidemiological data, other confounding factors affecting HPV and CIN (e.g., smoking) were also recorded. As stated, according to the policy, all the women were advised to proceed to HPV immunization regardless of their age. By extensively presenting benefits of the immunization, all medical teams tried to convince non-vaccinated women to carry on with a full 3-dose HPV vaccination with the 2-valent or the 4-valent vaccine. Individuals had to self-finance the vaccine regimen without being reimbursed; no arrangements were made to provide free vaccination to the participating women.

In all the women, just prior to the colposcopic evaluation, a liquid-based cytology (LBC) sample was obtained and sent for cytological and biomolecular analysis for established HPV-related biomarkers, in particular:

- HPV DNA genotyping (CLART-2 HPV test)
- mRNA E6 and E7 for high-risk HPV types 16, 18, 31, 33, and 45 [tested by the NASBA method].

The HPV DNA genotyping using CLART® HUMAN PAPILLOMAVIRUS 2 allows simultaneous detection of 35 different HPV genotypes (both high- and low-risk, including the subtypes included in this study) by PCR amplification of a fragment within the highly conserved L1 region of the virus [38], while the NASBA assay [39] (NucliSENS EasyQ® HPV v1.0) allows identification of E6/E7 mRNA of HPV types 16, 18, 31, 33, and 45 [40].

The cytological examination was expressed according to the Bethesda classification (TBS2001 system) [41,42]. As stated, all colposcopic examinations were performed by expert board-accredited colposcopists, one physician for each center. Women with low-grade referral LBC and low-grade colposcopic impression who tested HPV DNA-positive for one of the aforementioned 5 HR HPV subtypes were included in the study population independently of their mRNA E6 and E7 result; these women were asked to be prospectively followed up one year later.

At the follow-up visit, 12 months post-recruitment, all women’s history forms were updated regarding details focusing on aspects of sexual history (e.g., changes in sexual partnership, etc.). Women were asked if they had completed the full three-dose HPV vaccination regimen or not, commencing no later than 1-month post-recruitment. In this case, they were asked to provide specific information regarding the vaccine brand and a detailed administration calendar; all these data were recorded. Then, a new LBC vial was obtained and sent for cytological and biomolecular evaluation for the same HPV-related biomarkers as during the recruitment and subsequently underwent a repeat colposcopic examination. Women with disease progression (cytological and or colposcopic impression of ≥ LG) were scheduled for biopsies or treatment during a subsequent visit, outside the timeframe of the study.

**Figure 1:**
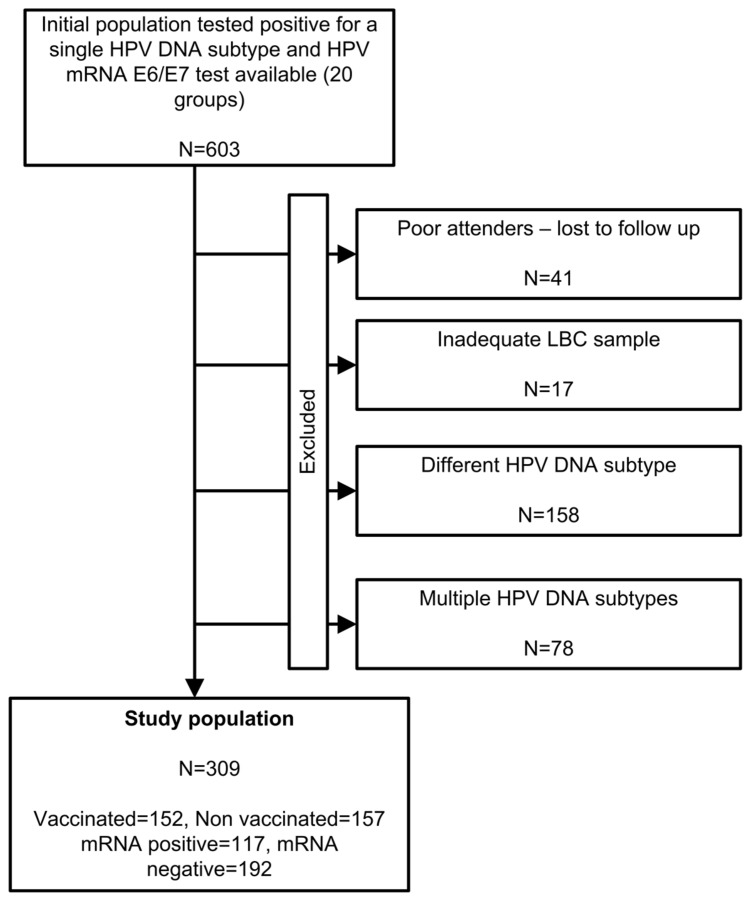
Study flowchart.

The flowchart of the study population is depicted in Figure 1; from the 603 women that initially tested positive for the studied HPV subtypes and were eligible to participate, 309 remained in the study until the end, since a high percentage (49%) of them discontinued for various reasons (see Figure 1).

### 4.4. Analysis

In order to explore the alterations of HPV-related biomarker positivity rates after HPV vaccination, we compared the vaccinated population with a matched control cohort, performing two groups of analyses.

The 1st analysis focused on those individuals who had HPV DNA-positive and mRNA-negative infections; these groups were vaccinated group 1 (V1) and non-vaccinated group 1 (N-V1).

The 2nd analysis group was based on the population that was found to have both HPV DNA test and the mRNA test positive at the first visit. The groups analyzed in the 2nd analysis were named: vaccinated group 2 (V2) and non-vaccinated group 2 (N-V2).

### 4.5. Statistical Methods and Tools

Statistical analysis was performed with the SAS for Windows 9.4 software platform (SAS Institute Inc., NC, USA). Descriptive values were expressed as the means ± standard deviation (SD) or percentages. Comparisons between groups in terms of the qualitative parameters were made using the chi-squared test. For the continuous parameters (woman’s age, age at the first sexual intercourse, and number of sexual partners), it was not possible to ensure normality, therefore, non-parametric tests were applied, specifically, the Kruskal–Wallis test. Furthermore, we calculated the power of the study for the cumulative results of HPV DNA and mRNA positivity rates in the vaccinated and non-vaccinated groups. The significance level (*p*-value) was set to 0.05, thus, the statistically significant difference between the compared groups was with *p* < 0.05. We assessed HPV DNA genotyping and mRNA E6 and E7 positivity rates after administration or not of the vaccine in theV1 and N-V1 groups as well as in the V2 and N-V2 groups. Accuracy parameters like the *p*-value, odds ratios (OD), relative risk (RR), absolute relative risk (ARR), and number needed to treat (NNT) with confidence intervals (CI 95%) were calculated for each group.

## 5. Conclusions

This study’s findings gain additional importance in the context of shared decision-making while offering the HPV vaccine to adults aged 27 to 45 years who have not started or completed the vaccine course recently advocated by a statement of the American Committee on Immunization Practices [43,44], especially since questions on HPV DNA positivity and HPV detection rates following HPV vaccination of older cohorts are among the most frequently enquired by candidate vaccine recipients before finalizing their informed decisions [44].

In patients with LG cytological abnormalities, carrying out an HPV16, HPV18, HPV31 and HPV33 VLP vaccination appeared to ensure in a statistically significant manner an earlier clearance of HPV infection in comparison with the patients included in the non-vaccination cohort. Regarding HPV mRNA clearance, a mild molecular reduction (negation) with no statistical significance was documented, with the exception of HPV31 mRNA E6/E7 which reached statistical significance. Despite the pilot character of our study in an emerging field, we strongly believe that larger patient cohorts and more robust data are necessary in order to elucidate which oncogene’s expression represents the most suitable biomarker to assess vaccines’ efficacy.

## Figures and Tables

**Table 1:** Distribution of the participating women according to HPV infection subtype across the study groups. V1: vaccinated women with HPV DNA-positive and mRNA-negative results, NV1: non-vaccinated women with HPV DNA-positive and mRNA-negative results, V2: vaccinated women with HPV DNA- and mRNA-positive results, NV2: non-vaccinated women with HPV DNA- and mRNA-positive results.

Group / HPV subtype	HPV16	HPV18	HPV31	HPV33	HPV45	Total
**V1**	24	25	28	18	4	**99**
**NV1**	28	25	20	17	3	**93**
**V2**	12	13	15	11	2	**53**
**NV2**	13	20	16	14	1	**64**
**Total**	**77**	**83**	**79**	**60**	**10**	**309**

**Table 2:** Demographic characteristics of the studied population, comparison between the vaccinated and non-vaccinated women and comparison between the HPV mRNA-positive and mRNA-negative women (all the individuals listed in this Table tested HPV DNA-positive at recruitment). SD: standard deviation.

Demographic Characteristics	All Groups	Vaccinated	Non-Vaccinated	*p* *	mRNA-positive	mRNA-negative	*p* *
**Population**	309	152	157		117	192	
**Age in years (mean ± SD)**	26.53 ± 5.16	26.41 ± 5.21	26.64 ± 5.14	0.6278	26.82 ± 5.22	26.35 ± 5.14	0.4607
**Parity (Yes/No) and %**	77/232 (24.92%, 75.08%)	36/116 (23.68%, 76.32%)	41/116 (26.11%, 73.89%)	0.6215	36/81 (30.77%, 69.23%)	41/151 (21.35%, 78.65%)	0.0635
**Smokers (Yes/No) and %**	161/148 (52.1%, 47.9%)	87/65 (57.24%, 42.76%)	74/83 (47.13%, 52.87%)	0.2368	66/51 (56.41%, 43.59%)	95/97 (49.48%, 50.52%)	0.2854
**Onset of sexual intercourses in years (mean ± SD)**	16.95 ± 1.97	16.94 ± 1.75	16.96 ± 2.18	0.6527	17.09 ± 2.15	16.86 ± 1.86	0.5213
**Number of sexual partners (mean ± SD)**	4.3 ± 2.67	4.45 ± 2.74	4.16 ± 2.61	0.3281	4.26 ± 2.72	4.33 ± 2.65	0.6823

* *P*-values indicate comparisons either between the observed arithmetic values or between the observed percentages in vaccinated and non-vaccinated women or in HPV mRNA-positive and HPV mRNA-negative women. In all the cases, no statistically significant differences were found between the studied groups, a fact indicating that the studied populations can be considered similar in terms of the characteristics reported in the first column of this table.

**Table 3:** Evolution of HPV-related infections in the study period depending on specific HPV genotype. Analytical results of the studied groups. All cases were positive for the specified HPV subtype (16, 18, 31, 33, or 45) before vaccination. N: number of cases within each study group, OR: odds ratio, RR: relative risk, ARR: absolute relative risk, NNT: number needed to treat, CI: confidence interval, V1: vaccinated women with HPV DNA-positive and mRNA-negative results, NV1: non-vaccinated women with HPV DNA-positive and mRNA-negative results, V2: vaccinated women with HPV DNA- and mRNA-positive results, NV2: non-vaccinated women with HPV DNA- and mRNA-positive results.

HPV Genotype (N, %)	Group	N	HPV DNA+ at 12 Months (N, %)	HPV DNA Clearance (%)	HPV mRNA+ at 12 Months (N, %)	*p*-value *	OR (95% CI)	RR (95% CI)	ARR (95% CI)	NNT (95% CI)
**16 (77, 24.92%)**	**V1**	24	4, 16.67%	83.33%	0, 0%	**0.043**	4.333 (1.175 to 15.986)	1.556 (1.055 to 2.294)	−0.298 (−0.501 to −0.042)	−3 (−2 to −24)
	**NV1**	28	13, 46.43%	53.57%	3, 10.71%					
	**V2**	12	6, 50%	50.00%	4, 33.33%	0.567	2.25 (0.439 to 11.522)	1.625 (0.602 to 4.384)	−0.192 (−0.498 to 0.172)	−5 (−2 to −6)
	**NV2**	13	9, 69.23%	30.77%	7, 53.85%					
**18 (83, 26.86%)**	**V1**	25	6, 24%	76.00%	1, 4%	**0.043**	4.03 (1.201 to 13.526)	1.727 (1.054 to 2.831)	−0.12 (−0.534 to −0.049)	−3 (−2 to −21)
	**NV1**	25	14, 56%	44.00%	3, 12%					
	**V2**	13	6, 46.15%	53.85%	3, 23.08%	0.672	1.75 (1.006 to 21.653)	1.346 (0.645 to 2.81)	−0.138 (−0.431 to 0.188)	−7 (−2 to −5)
	**NV2**	20	12, 60%	40.00%	7, 35%					
**31 (79, 25.57%)**	**V1**	28	6, 21.43%	78.57%	3, 10.71%	**0.036**	4.481 (1.27 to 15.82)	1.746 (1.036 to 2.942)	−0.336 (−0.558 to −0.06)	−3 (−2 to −17)
	**NV1**	20	11, 55%	45.00%	7, 35%					
	**V2**	15	8, 53.33%	46.67%	4, 26.67%	0.879	0.0481 (0.0025 to 0.9314)	1.244 (0.541 to 2.861)	−0.092 (−0.392 to 0.232)	−11 (−3 to 4)
	**NV2**	16	10, 62.5%	37.50%	8, 50%					
**33 (60, 19.42%)**	**V1**	18	5, 27.78%	72.22%	2, 11.11%	0.407	2.311 (0.568 to 9.408)	1.364 (0.801 to 2.322)	−0.193 (−0.46 to 0.119)	−5 (−2 to 8)
	**NV1**	17	8, 47.06%	52.94%	3, 17.65%					
	**V2**	11	5, 45.45%	54.55%	5, 45.45%	0.859	1.6 (0.3262 to 7.848)	1.273 (0.566 to 2.862)	−0.117 (−0.44 to 0.245)	−9 (−2 to 4)
	**NV2**	14	8, 57.14%	100.00%	5, 35.71%					
**45 (10, 3.24%)**	**V1**	4	0, 0%	100.00%	1, 25%	0.343	1.2857 (0.02 to 82.497)	1.029 (0.642 to 1.649)	−0.025 (−0.513 to 0.426)	−40 (−2 to 2)
	**NV1**	3	0, 0%	100.00%	1, 33.33%					
	**V2**	2	1, 50%	50.00%	1, 50%	0.665	0.333 (0.0066 to 16.80)	0.5 (0.125 to 0.905)	0.5 (−0.391 to 0.905)	2 (−3 to 1)
	**NV2**	1	0, 0%	100.00%	0, 0%					

* *P*-values indicate results of the chi-squared test of the HPV DNA-positive cases before and after vaccination, statistically significant differences are indicated in bold. A significant reduction in HPV DNA positivity rates was observed in vaccinated individuals only in the V1 Groups (HPV DNA (+) and HPV mRNA (-)] for HPV genotypes 16, 18, and 31.

**Table 4:** HPV DNA status at the end of the study for the overall population grouped into vaccinated and non-vaccinated women. For each group, the top row of each cell represents the number of cases, while the lower row represents the percentage of cases being HPV DNA-negative or positive.

Vaccination Status	HPV DNA Status at the End of the Study		
	Negative	Positive	Total
**Non-vaccinated**	72	85	157
	45.90%	54.10%	
**Vaccinated**	105	47	152
	69.10%	30.90%	
**Total**	177	132	309
	57.30%	42.70%	100%

**Table 5:** HPV mRNA status at the end of the study for the overall population grouped into vaccinated and non-vaccinated women. For each group, the top row of each cell represents the number of cases, while the lower row represents the percentage of cases being HPV mRNA-negative or positive.

Vaccination Status	HPV mRNA Status at the End of the Study		
	Negative	Positive	Total
**Non-vaccinated**	37	27	64
	57.8%	42.2%	
**Vaccinated**	36	17	53
	67.9%	32.1%	
**Total**	73	44	117
	62.4%	37.6%	100%

## References

[1] Simms K.T., Steinberg J., Caruana M., Smith M.A., Lew J.B., Soerjomataram I., Castle P.E., Bray F., Canfell K. (2019). Impact of scaled up human papillomavirus vaccination and cervical screening and the potential for global elimination of cervical cancer in 181 countries, 2020–2099: A modelling study. Lancet Oncol..

[2] Luostarinen T., Apter D., Dillner J., Eriksson T., Harjula K., Natunen K., Paavonen J., Pukkala E., Lehtinen M. (2018). Vaccination protects against invasive HPV-associated cancers. Int. J. Cancer.

[3] Tsakiroglou M., Bakalis M., Valasoulis G., Paschopoulos M., Koliopoulos G., Paraskevaidis E. (2011). Women’s knowledge and utilization of gynecological cancer prevention services in the Northwest of Greece. Eur. J. Gynaecol. Oncol..

[4] Valasoulis G., Stasinou S., Kyrgiou M., Arbyn M., Loufopoulos A., Nasioutziki M., Daponte A., Karakitsos P., Paraskevaidis E. An Update on Alterations of HPV-Related Biomarkers After Prophylactic HPV Vaccination. Proceedings of the Annual Scientific Meeting of the British Society for Colposcopy and Cervical Pathology.

[5] Giuliano A.R., Lazcano-Ponce E., Villa L., Nolan T., Marchant C., Radley D., Golm G., McCarroll K., Yu J., Esser M.T. (2007). Impact of baseline covariates on the immunogenicity of a quadrivalent (types 6, 11, 16, and 18) human papillomavirus virus-like-particle vaccine. J. Infect. Dis..

[6] Stanley M. (2010). HPV—immune response to infection and vaccination. Infect. Agents Cancer.

[7] Hildesheim A., Herrero R., Wacholder S., Rodriguez A.C., Solomon D., Bratti M.C., Schiller J.T., Gonzalez P., Dubin G., Porras C. (2007). Effect of human papillomavirus 16/18 L1 viruslike particle vaccine among young women with preexisting infection: A randomized trial. JAMA J. Am. Med. Assoc..

[8] Mollers M., King A.J., Knol M.J., Scherpenisse M., Meijer C.J., van der Klis F.R., de Melker H.E. (2015). Effectiveness of human papillomavirus vaccine against incident and persistent infections among young girls: Results from a longitudinal Dutch cohort study. Vaccine.

[9] Fu T.C., Carter J.J., Hughes J.P., Feng Q., Hawes S.E., Schwartz S.M., Xi L.F., Lasof T., Stern J.E., Galloway D.A. (2016). Re-detection vs. new acquisition of high-risk human papillomavirus in mid-adult women. Int. J. Cancer. J. Int. Du Cancer.

[10] U.S. FDA Package Insert—GARDASIL 9. https://www.fda.gov/media/90064/download.

[11] Bosch F.X., Robles C., Diaz M., Arbyn M., Baussano I., Clavel C., Ronco G., Dillner J., Lehtinen M., Petry K.U. (2016). HPV-FASTER: Broadening the scope for prevention of HPV-related cancer. Nat. Rev. Clin. Oncol..

[12] Vorsters A., Van Damme P., Bosch F.X. (2019). HPV vaccination: Are we overlooking additional opportunities to control HPV infection and transmission?. Int. J. Infect. Dis..

[13] Joura E.A., Garland S.M., Paavonen J., Ferris D.G., Perez G., Ault K.A., Huh W.K., Sings H.L., James M.K., Haupt R.M. (2012). Effect of the human papillomavirus (HPV) quadrivalent vaccine in a subgroup of women with cervical and vulvar disease: Retrospective pooled analysis of trial data. BMJ.

[14] Kang W.D., Choi H.S., Kim S.M. (2013). Is vaccination with quadrivalent HPV vaccine after loop electrosurgical excision procedure effective in preventing recurrence in patients with high-grade cervical intraepithelial neoplasia (CIN2-3)?. Gynecol. Oncol..

[15] Garland S.M., Paavonen J., Jaisamrarn U., Naud P., Salmeron J., Chow S.N., Apter D., Castellsague X., Teixeira J.C., Skinner S.R. (2016). Prior human papillomavirus-16/18 AS04-adjuvanted vaccination prevents recurrent high grade cervical intraepithelial neoplasia after definitive surgical therapy: Post-hoc analysis from a randomized controlled trial. Int. J. Cancer. J. Int. Du Cancer.

[16] Ghelardi A., Parazzini F., Martella F., Pieralli A., Bay P., Tonetti A., Svelato A., Bertacca G., Lombardi S., Joura E.A. (2018). SPERANZA project: HPV vaccination after treatment for CIN2. Gynecol. Oncol..

[17] Arbyn M., Tommasino M., Depuydt C., Dillner J. (2014). Are 20 human papillomavirus types causing cervical cancer?. J. Pathol..

[18] ICO/IARC Information Centre on HPV and Cancer Greece, Human Papillomavirus and Related Cancers, Fact Sheet 2018. https://hpvcentre.net/statistics/reports/GRC_FS.pdf?t=1586602338630.

[19] Valasoulis G., Koliopoulos G., Founta C., Kyrgiou M., Tsoumpou I., Valari O., Martin-Hirsch P., Daponte A., Karakitsos P., Paraskevaidis E. (2011). Alterations in human papillomavirus-related biomarkers after treatment of cervical intraepithelial neoplasia. Gynecol. Oncol..

[20] Cordeiro Valenca J.E., Goncalves A.K., Cotrim Guerreiro da Silva I.D., Eleuterio Junior J., Buarque Valenca C., Buarque Valenca T., Bezerra Menezes M.L., Arraes de Alencar Ximenes R. (2017). The influence of biopsy in cervical high-grade squamous intraepithelial lesion, evaluated by HPV E6/E7 mRNA, Pap test, and conization results. Eur. J. Gynaecol. Oncol..

[21] Kyrgiou M., Pouliakis A., Panayiotides J.G., Margari N., Bountris P., Valasoulis G., Paraskevaidi M., Bilirakis E., Nasioutziki M., Loufopoulos A. (2016). Personalised management of women with cervical abnormalities using a clinical decision support scoring system. Gynecol. Oncol..

[22] Valasoulis G., Stasinou S.M., Nasioutziki M., Athanasiou A., Zografou M., Spathis A., Loufopoulos A., Karakitsos P., Paraskevaidis E., Kyrgiou M. (2014). Expression of HPV-related biomarkers and grade of cervical intraepithelial lesion at treatment. Acta Obstet. Gynecol. Scand..

[23] Wheeler C.M., Castellsague X., Garland S.M., Szarewski A., Paavonen J., Naud P., Salmeron J., Chow S.N., Apter D., Kitchener H. (2012). Cross-protective efficacy of HPV-16/18 AS04-adjuvanted vaccine against cervical infection and precancer caused by non-vaccine oncogenic HPV types: 4-year end-of-study analysis of the randomised, double-blind PATRICIA trial. Lancet Oncol..

[24] Latsuzbaia A., Arbyn M., Tapp J., Fischer M., Weyers S., Pesch P., Mossong J. (2019). Effectiveness of bivalent and quadrivalent human papillomavirus vaccination in Luxembourg. Cancer Epidemiol..

[25] Arbyn M., Xu L., Simoens C., Martin-Hirsch P.P. (2018). Prophylactic vaccination against human papillomaviruses to prevent cervical cancer and its precursors. Cochrane Database Syst. Rev..

[26] Artemchuk H., Eriksson T., Poljak M., Surcel H.M., Dillner J., Lehtinen M., Faust H. (2019). Long-term Antibody Response to Human Papillomavirus Vaccines: Up to 12 Years of Follow-up in the Finnish Maternity Cohort. J. Infect. Dis..

[27] Draper E., Bissett S.L., Howell-Jones R., Waight P., Soldan K., Jit M., Andrews N., Miller E., Beddows S. (2013). A randomized, observer-blinded immunogenicity trial of Cervarix((R)) and Gardasil((R)) Human Papillomavirus vaccines in 12-15 year old girls. PLoS ONE.

[28] Einstein M.H., Takacs P., Chatterjee A., Sperling R.S., Chakhtoura N., Blatter M.M., Lalezari J., David M.P., Lin L., Struyf F. (2014). Comparison of long-term immunogenicity and safety of human papillomavirus (HPV)-16/18 AS04-adjuvanted vaccine and HPV-6/11/16/18 vaccine in healthy women aged 18-45 years: End-of-study analysis of a Phase III randomized trial. Hum. Vaccines Immunother..

[29] Daponte A., Pournaras S., Mademtzis I., Hadjichristodoulou C., Kostopoulou E., Maniatis A.N., Messinis I.E. (2006). Evaluation of HPV 16 PCR detection in self- compared with clinician-collected samples in women referred for colposcopy. Gynecol. Oncol..

[30] Daponte A., Pournaras S., Mademtzis I., Hadjichristodoulou C., Kostopoulou E., Maniatis A.N., Messinis I.E. (2006). Evaluation of high-risk human papillomavirus types PCR detection in paired urine and cervical samples of women with abnormal cytology. J. Clin. Virol..

[31] Daponte A., Tsezou A., Oikonomou P., Hadjichristodoulou C., Maniatis A.N., Pournaras S., Messinis I.E. (2008). Use of real-time PCR to detect human papillomavirus-16 viral loads in vaginal and urine self-sampled specimens. Clin. Microbiol. Infect..

[32] Dardiotis E., Siokas V., Garas A., Paraskevaidis E., Kyrgiou M., Xiromerisiou G., Deligeoroglou E., Galazios G., Kontomanolis E.N., Spandidos D.A. (2018). Genetic variations in the SULF1 gene alter the risk of cervical cancer and precancerous lesions. Oncol. Lett..

[33] Daponte A., Grayson W., Moisuc D., Ebrahim S., Guidozzi F. (2003). Adenoid cystic carcinoma stage Ib1 treated with radical surgery displaying human papilloma virus 33 (HPV 33): Immunoelectron microscopy and review. Gynecol. Oncol..

[34] Agorastos T., Chatzistamatiou K., Tsertanidou A., Mouchtaropoulou E., Pasentsis K., Kitsou A., Moysiadis T., Moschaki V., Skenderi A., Katsiki E. (2019). Implementation of HPV-based Cervical Cancer Screening Combined with Self-sampling Using a Midwifery Network Across Rural Greece: The GRECOSELF Study. Cancer Prev. Res..

[35] Tsoumpou I., Valasoulis G., Founta C., Kyrgiou M., Nasioutziki M., Daponte A., Koliopoulos G., Malamou-Mitsi V., Karakitsos P., Paraskevaidis E. (2011). High-risk human papillomavirus DNA test and p16(INK4a) in the triage of LSIL: A prospective diagnostic study. Gynecol. Oncol..

[36] Valari O., Koliopoulos G., Karakitsos P., Valasoulis G., Founta C., Godevenos D., Dova L., Paschopoulos M., Loufopoulos A., Paraskevaidis E. (2011). Human papillomavirus DNA and mRNA positivity of the anal canal in women with lower genital tract HPV lesions: Predictors and clinical implications. Gynecol. Oncol..

[37] Tamposis I., Iordanidis E., Tzortzis L., Bountris P., Haritou M., Koutsouris D., Pouliakis A., Karakitsos P. HPVGuard: A software platform to support management and prognosis of cervical cancer. Proceedings of the 2014 4th International Conference on Wireless Mobile Communication and Healthcare - Transforming Healthcare Through Innovations in Mobile and Wireless Technologies (MOBIHEALTH).

[38] Gomez-Roman J.J., Echevarria C., Salas S., Gonzalez-Moran M.A., Perez-Mies B., Garcia-Higuera I., Nicolas Martinez M., Val-Bernal J.F. (2009). A type-specific study of human papillomavirus prevalence in cervicovaginal samples in three different Spanish regions. APMIS.

[39] Tyagi S., Bratu D.P., Kramer F.R. (1998). Multicolor molecular beacons for allele discrimination. Nat. Biotechnol..

[40] Koliopoulos G., Valasoulis G., Zilakou E. (2009). An update review on HPV testing methods for cervical neoplasia. Expert Opin. Med. Diagn..

[41] Henry M.R. (2003). The Bethesda System 2001: An update of new terminology for gynecologic cytology. Clin. Lab. Med..

[42] Smith J.H. (2002). Bethesda 2001. Cytopathol. Off. J. Br. Soc. Clin. Cytol..

[43] Meites E., Szilagyi P.G., Chesson H.W., Unger E.R., Romero J.R., Markowitz L.E. (2019). Human Papillomavirus Vaccination for Adults: Updated Recommendations of the Advisory Committee on Immunization Practices. MMWR Morb. Mortal. Wkly. Rep..

[44] Oshman L.D., Davis A.M. (2020). Human Papillomavirus Vaccination for Adults: Updated Recommendations of the Advisory Committee on Immunization Practices (ACIP). JAMA J. Am. Med. Assoc..

